# 

**Published:** 1946

**Authors:** 


### CONTENTS.

| | Page |
|---|---|
| The Diagnosis of Tumours of the Central Nervous System. By Diana J. Kinloch Beck, M.B., B.S., F.R.C.S., F.R.C.S.E. .. | 65 |
| Diagnosis of Tumours of the Central Nervous System. By Hugh Cairns, D.M., F.R.C.S. .. .. .. .. .. | 73 |
| Dioscorides Phakas on the Detection of Iron in Blood, Urine and Sputum. By M. Nierenstein, D.Sc. .. .. .. .. | 75 |
| Inhalation of Food during Injection of Pentothal. By R. Hastings Moore, M.B., Ch.B., M.R.C.S., L.R.C.P., D.A. .. .. .. | 82 |
| Reviews of Books .. .. .. .. .. .. .. .. | 84 |
| The Medical Library of the University of Bristol .. .. .. | 86 |
| Publications Received .. .. .. .. .. .. .. | 87 |
| Local Medical Notes .. .. .. .. .. .. .. | 88 |

